# Liquid to Use in Porcelain Work

**Published:** 1904-08

**Authors:** J. L. Helmer

**Affiliations:** Kokomo, Ind.


### LIQUID TO USE IN PORCELAIN WORK.

By J. L. Helmer, D. D. S.,

Kokomo, Ind.

(Written for *The American Journal of Dental Science.*)

Alcohol I have found is the best liquid with which to moisten the porcelain powder, for the evaporation takes place more rapidly and there is no steam formed; I attribute bubbles to the steam that is formed after the case is placed in the furnace. I may be wrong on this point, but anyway the rapid evaporation of the alcohol allows one to pack the powder tighter and when the case is inserted in the furnace there is not enough moisture to make pieces of the body pop off. I have found that I can work porcelain bodies with pure alcohol easier than I can with any other liquid. It will do no harm for you to try this and it may work better than what you are now using with which to moisten the porcelain powder. In a piece of porcelain in which there is a bubble of any perceptible size, the desired shade is not obtained because of this bubble the shade is thrown off to some extent. So in the working of porcelain an aim should be to obtain a dense piece as free from bubbles as possible.

### MIXING OF CEMENT FOR SETTING INLAYS.

The mixing of the cement for the setting of inlays is a great feature of the inlay problem. At different times I have made inlays for cavities in extracted teeth—just to keep my hand in training—and found that they were very easily fractured out, some time after being cemented in the cavity. Why was this? I did not mix the cement properly. I had been told and we all have been told to mix the cement to a creamy consistency. I had done that and the inlays were easily fractured out just the same. But I came on to it a short time ago how to better mix my cement so that it will hold the inlay better. I think one of the great troubles inlay workers have is in the fact of the inlays not staying in place after being cemented.

Cement usually is mixed too rapidly, the ingredients are not thoroughly incorporated. Now, don't mix cement so rapidly, take your time at it. Incorporate a little of the powder with the liquid, thoroughly mixing them before adding any more of the powder. When the first bit of the powder is thoroughly mixed then add a little bit more, mixing it in thoroughly. Continue to add powder little by little, thoroughly mixing and spatulating till you have a mix some thicker than cream. Cement mixed this way will not crystalize rapidly. The adhesive properties are much greater than when mixed rapidly.

